# Pasture Feeding Changes the Bovine Rumen and Milk Metabolome

**DOI:** 10.3390/metabo8020027

**Published:** 2018-04-06

**Authors:** Tom F. O’Callaghan, Rosa Vázquez-Fresno, Arnau Serra-Cayuela, Edison Dong, Rupasri Mandal, Deirdre Hennessy, Stephen McAuliffe, Pat Dillon, David S. Wishart, Catherine Stanton, R. Paul Ross

**Affiliations:** 1 Teagasc Food Research Centre, Moorepark, Fermoy, P61 C996 Cork, Ireland; tom.ocallaghan@teagasc.ie (T.F.O.); catherine.stanton@teagasc.ie (C.S.); 2 APC Microbiome Institute, University College Cork, T12 YT20 Cork, Ireland; 3 The Metabolomics Innovation Centre (TMIC), University of Alberta, Edmonton, AB T6G1C9, Canada; vazquezf@ualberta.ca (R.V.-F.); serracay@ualberta.ca (A.S.-C.); ywdong@ualberta.ca (E.D.); rmandal@ualberta.ca (R.M.); dwishart@ualberta.ca (D.S.W.); 4 Teagasc Animal & Grassland Research and Innovation Centre, Moorepark, Fermoy, P61 C996 Cork, Ireland; Deirdre.Hennessy@teagasc.ie (D.H.); Stephen.McAuliffe@teagasc.ie (S.M.); Pat.Dillon@teagasc.ie (P.D.); 5 School of Biological Sciences, Queens University, Belfast BT7 1NN, Northern Ireland, UK; 6 College of Science, Engineering and Food Science, University College Cork, T12 YT20 Cork, Ireland

**Keywords:** cows diet, rumen, milk, metabolome, pasture, total mixed ration

## Abstract

The purpose of this study was to examine the effects of two pasture feeding systems—perennial ryegrass (GRS) and perennial ryegrass and white clover (CLV)—and an indoor total mixed ration (TMR) system on the (a) rumen microbiome; (b) rumen fluid and milk metabolome; and (c) to assess the potential to distinguish milk from different feeding systems by their respective metabolomes. Rumen fluid was collected from nine rumen cannulated cows under the different feeding systems in early, mid and late lactation, and raw milk samples were collected from ten non-cannulated cows in mid-lactation from each of the feeding systems. The microbiota present in rumen liquid and solid portions were analysed using 16S rRNA gene sequencing, while ^1^H-NMR untargeted metabolomic analysis was performed on rumen fluid and raw milk samples. The rumen microbiota composition was not found to be significantly altered by any feeding system in this study, likely as a result of a shortened adaptation period (two weeks’ exposure time). In contrast, feeding system had a significant effect on both the rumen and milk metabolome. Increased concentrations of volatile fatty acids including acetic acid, an important source of energy for the cow, were detected in the rumen of TMR and CLV-fed cows. Pasture feeding resulted in significantly higher concentrations of isoacids in the rumen. The ruminal fluids of both CLV and GRS-fed cows were found to have increased concentrations of *p*-cresol, a product of microbiome metabolism. CLV feeding resulted in increased rumen concentrations of formate, a substrate compound for methanogenesis. The TMR feeding resulted in significantly higher rumen choline content, which contributes to animal health and milk production, and succinate, a product of carbohydrate metabolism. Milk and rumen-fluids were shown to have varying levels of dimethyl sulfone in each feeding system, which was found to be an important compound for distinguishing between the diets. CLV feeding resulted in increased concentrations of milk urea. Milk from pasture-based feeding systems was shown to have significantly higher concentrations of hippuric acid, a potential biomarker of pasture-derived milk. This study has demonstrated that ^1^H-NMR metabolomics coupled with multivariate analysis is capable of distinguishing both rumen-fluid and milk derived from cows on different feeding systems, specifically between indoor TMR and pasture-based diets used in this study.

## 1. Introduction

Traditionally, a dairy cow’s natural diet has consisted of fresh grasses and forages grazed outdoors which, in countries such as Ireland and New Zealand, is still the practice for the majority of the cow’s lactation period. In recent decades, the intensification of the dairy industry has resulted in a shift to more conventional total mixed ration (TMR) feeding systems where cows are housed indoors year-round and fed a diet of grass and maize silage supplemented with high levels of concentrates. TMR feeding is widely practiced in the US, parts of Europe, the Middle East, and Asia. Indeed, the United States Department of Agriculture reported that in 2014, <7% of dairy operations in the US were grazing only based systems [1]. Total mixed ration feeding systems allow the farmer greater control over the cow’s nutrition and also offers the animals protection from environmental extremes [2]. In contrast, pasture feeding systems allow the animals access to fresh forages and enable them to perform typical behaviors in a natural environment such as grazing [3].

There is an increased demand by consumers for pasture-derived dairy products, resulting from consumer perceptions of a healthier, more natural product and improved animal welfare compared to the more conventional indoor TMR feeding systems [4]. These perceptions appear to have some basis in fact. O’Callaghan et al. [5] demonstrated that pasture derived milk has increased protein quality, and an improved fatty acid profile with significantly higher conjugated linoleic acid (CLA) and omega 3 fatty acid content than milk derived from a TMR feeding system throughout lactation. As such, “pasture-based” dairy has become a major part of dairy marketing schemes in countries which use them, such as New Zealand and Ireland. However, there is limited information and essentially no method currently available for the verification of such pasture-derived dairy products and their source of primary production.

Quantitative nuclear magnetic resonance (^1^H-NMR) has been successfully used in the past to investigate and characterize both the rumen [6] and milk [7] metabolomes. ^1^H-NMR is an attractive method for metabolomic analysis as it requires minimal sample preparation, low sample volumes, and is by nature a non-targeted approach [8]. A study by Sundekilde et al. [8] comprehensively reviewed the many applications of ^1^H-NMR for milk analysis which include milk authentication and milk nutritional quality research.

Interestingly, using ^1^H-NMR spectroscopy, Ametaj et al. [9] revealed unhealthy alterations of the rumen metabolome with increasing proportions of cereal grain, and highlighted that different dietary systems could be distinguished based on the rumen metabolomic profile. Indeed, the effect of TMR feeding systems with increasing levels of cereal grain in the diet have been reported to result in unhealthy alterations to the rumen metabolome with increased concentrations of biogenic amines, such as methylamines and putrescine and increased rumen acidification [10]. However, there is limited information currently available comparing the effects of grazing pasture systems and TMR feeding systems on a cow’s ruminal fluid metabolome and raw milk metabolome.

The primary objectives of this study were to investigate the effects of three widely practiced feeding systems—consisting of a TMR diet indoors, perennial ryegrass (*Lolium perenne* L.) outdoors (GRS), and perennial ryegrass/white clover (*Trifolium repens* L.) outdoors (CLV) in Ireland—on (a) the cow’s rumen microbiome and (b) ruminal fluid and milk metabolome; and (c) to determine if it is possible to distinguish pasture derived milk from TMR produced milk using NMR-based metabolomics.

## 2. Results and Discussion

### 2.1. Effect of Cow Diet on Rumen Microbiota

**Figure 1:**
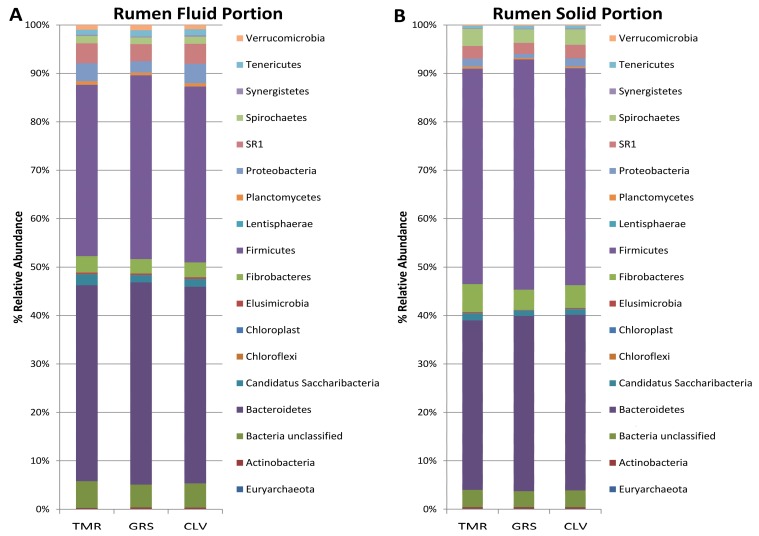
Bar chart demonstrating breakdown of abundances of different phyla in rumen fluid (**A**) and rumen solid (**B**) portions of rumen microbiota.

**Figure 2:**
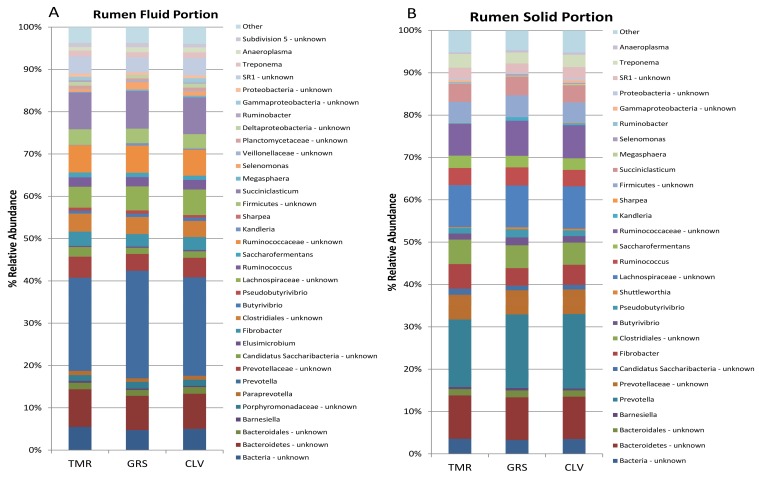
Bar chart demonstrating breakdown of abundances of different genera in rumen fluid (**A**) and rumen solid (**B**) portions of rumen microbiota. (Genera present at > 1% abundance in at least 1 sample).

DNA extracted from rumen fluid and solid portions was analysed using 16S rRNA gene sequencing initially, to determine if pasture feeding resulted in significant alteration of the rumen microbiota compared to TMR feeding. The dominant phyla in each of the rumen portions for each of the diets are shown in Figure 1. *Bacteroidetes* and *Firmicutes* have been reported previously as being the dominant phyla in the rumen [11,12], and their different abundances between the solid and liquid portions has been attributed to the biological functions associated with each phyla [13]. Indeed, the bacteria attached to the ingested plant matter within the rumen are thought to be responsible for initial fiber degradation [14,15]. The major genera for each of the diets in each portion of the rumen is shown in Figure 2. The major fully classified genera in each of the portions were *Prevotella, Succiniclasticum Fibrobacter*, *Ruminococcus*, and *Butyrivibrio*. Robert [16] also reported a similar core microbiota present in the rumen and noted that these microbes likely contributed to the basic function of the rumen microbial ecosystem. *Prevotella* has previously been reported as the most abundant genera in the rumen [16,17,18], and as such has been suggested to play a fundamental role in the rumen ecosystems [11]. *Prevotella* strains have been shown to be capable of producing propionate; thus increased levels of *Prevotella* in the rumen could therefore be beneficial in the goal to reduce agricultural levels of methane emission as they divert H_2_ away from methanogenesis [19,20].

There was no significant effect of diet on the rumen microbiota composition observed in this study. Although diet has previously been reported to have a significant effect on the rumen microbiota, the lack of significant effect with adjusted *p*-values in this study could be attributed to the short time period (two weeks) the cows were exposed to each of the diets. It has been previously reported that 4 to 12 days is adequate time for animals to adapt to a diet. Indeed for similar studies investigating the rumen metabolome an 11-day period was used [10]. While such a time period has been shown to be sufficient to alter the rumen metabolome, it may not have been long enough to alter the macro-composition of the rumen microbiota. Instead, this time period appeared to alter the functionality of the micro-organisms present. Previous studies analysing the effect of diet on the rumen microbiota have used dietary exposure periods of up to one month prior to sample analysis [11]. Therefore, it is noteworthy that, for future studies utilizing a similar adaptation time period, more in-depth microbiota analysis techniques such as SHOTGUN sequencing or meta-transcriptomics would be more appropriate than 16S DNA sequencing to demonstrate diet induced alterations to gene distribution and expression, which are likely responsible for the changes in the rumen and milk metabolome (described later).

Our study has demonstrated a high bacterial diversity in the rumen of animals fed pasture or concentrate diets (Figure 2). Up to 173 genera were detected, however, 53 of these were unknown and only 18 genera were fully classified and present at concentrations of at least 1% in at least one sample. An interesting aspect of the data has highlighted that, while there has been significant work done characterizing the rumen microbiota in the past, there is still a considerable number of unclassified genera present; in this study, on average 51.4% of the liquid portion and 56.1% of abundances in the solid portion accounted for unknown genera. This level of unclassified and unknown organisms is potentially due to the highly anaerobic environment of the rumen. As such, many of the organisms have previously been uncultivable, however, with improving anaerobic techniques and methodology becoming available, it is obvious that there are still considerable numbers of novel genera in this complex ecosystem yet to be discovered.

Stage of lactation (SOL) was shown to have a minor effect on the rumen microbiota in this study. Among the rumen fluid portion, SOL had a significant effect on the genera *Succiniclasticum* (*p* = 0.019), *Megasphaera* (*p* = 0.038), and an unknown genus of *Alphaproteobacteria* (*p* = 0.001) which was present in all samples at >1% of total abundances. Among the rumen-solid microbiota, SOL had a significant effect on the genera *Succiniclasticum* (*p* = 0.013) and *Megasphaera* (*p* = 0.020). *Succiniclasticum* was higher in early and mid-lactation (*p* < 0.001) than in late lactation samples. *Succiniclasticum* has previously been reported as one of the most abundant genera in the rumen [21,22], and has been identified as an important bacteria due to its ability to convert succinate to propionate [23]. Rumen *Megasphaera* was higher (*p* < 0.05) in early lactation than mid and late lactation. *Megasphaera* has previously been reported as a genus associated with adaptation of the ruminal community to low pH [24]. In particular, among the *Megasphaera* genera, *Megasphaera elsdenii* has received much attention for its ability to utilize lactic acid and prevent its accumulation; thus preventing lactic acidosis, and may have potential functional use in controlling rumen acidosis [25]. On review of the topic, Plaizier et al. [26] reported that early lactation cows are at higher risk of experiencing ruminal acidosis as a result of reduced ruminal absorption capacity for acids due to a reduction in the length and density of rumen papillae. Noel et al. [12] and Jewell et al. [27] also reported only small differences in the rumen bacterial communities over time. Noel et al. [12] attributed the small changes in rumen microbiota over lactation to changing pasture feed quality throughout the seasons of a lactation, and also the potential alterations as a result of the production phase of the cow. Interestingly, it was also highlighted that bacterial community structure returned close to the community structure associated with the same season in the yearly cycle, and it was concluded that the rumen bacterial community in a production herd is remarkably stable over time [12].

### 2.2. Rumen Fluid Metabolome

The ^1^H-NMR analysis of the ruminal fluids identified 63 metabolites present in each of the samples. Table 1 shows the average concentration of each of the metabolites in rumen fluid of cows fed TMR, GRS, and CLV. The average metabolite concentration for each feeding system at the different stages of lactation is shown in Supplementary Materials Table S1. The most abundant compounds present in the ruminal fluid metabolome were volatile/short chain fatty acids including propionate, butyrate, acetic acid, valerate, isobutyric acid, and isovaleric acid. Similar trends were also reported by Saleem et al. [6] who characterized the bovine rumen fluid metabolome using a variety of analytical technologies.

**Figure 3:**
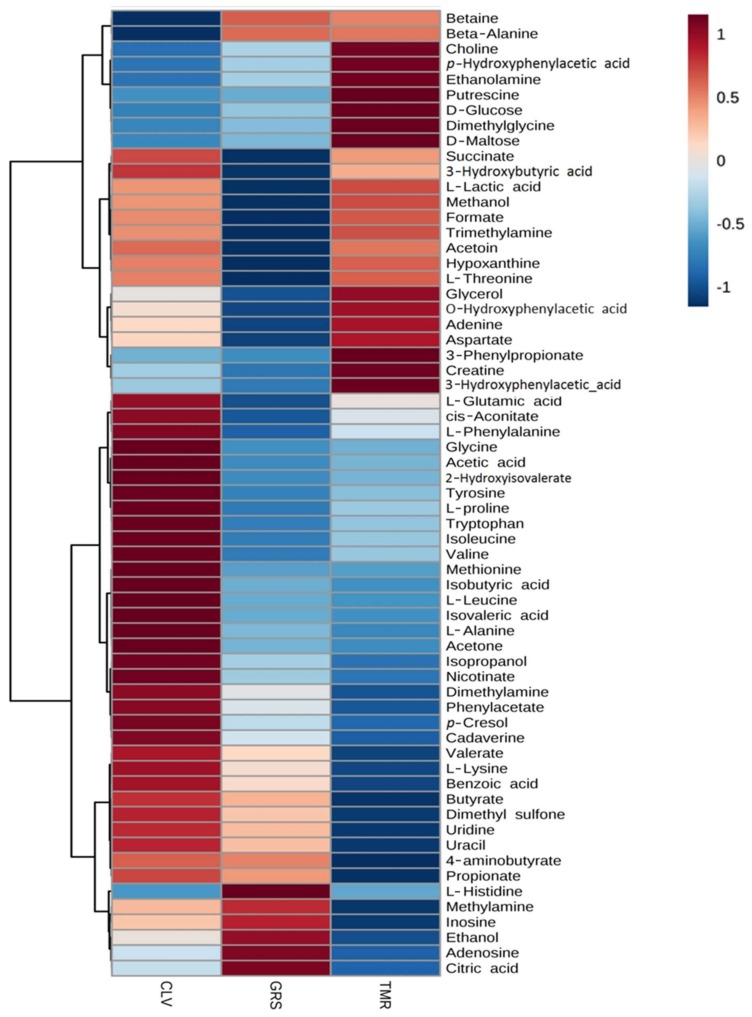
Hierarchical clustering analysis (heatmap) of average rumen fluid metabolites from lactating dairy cows fed diets consisting of total mixed ration (TMR), perennial ryegrass (GRS), or perennial ryegrass and white clover (CLV), as determined by ^1^H-NMR. The degree of positive and negative correlation of metabolite to dietary system is indicated by +1 (red) to −1 (blue).

**Figure 4:**
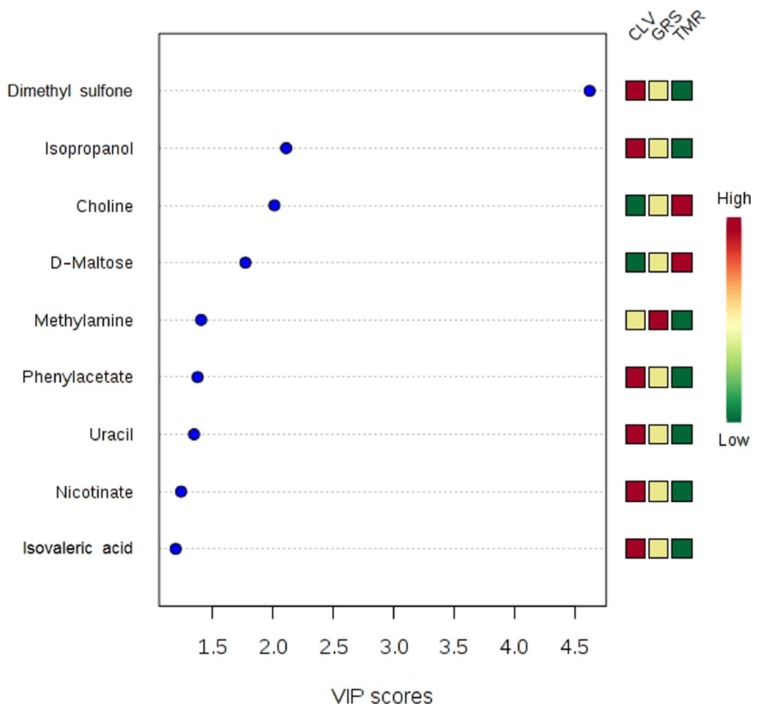
Variable importance plot (VIP) which shows the compounds primarily responsible for separation of rumen fluid metabolomes from cows fed diets consisting of total mixed ration (TMR), perennial ryegrass (GRS), or perennial ryegrass and white clover (CLV), as determined by PLS-DA.

The feeding system had a significant effect on the rumen metabolome as observed in the ANOVA test (diet *p*-values in Table 1). Such differences can be clearly observed in the clusters generated in the heatmap plot by the hierarchical clustering analysis (HCA) (Figure 3). It can be observed that the TMR group has higher levels of sugars, while the CLV group exhibits higher amino acid levels. The GRS group has a medium level of amino acids and high levels of nucleosides such as inosine and adenosine. The multivariate PLS-DA model (Supplementary Materials Figure S1) showed that it is possible to distinguish each of the feeding systems from each other using ^1^H-NMR analysis of the rumen fluid. While the ruminal fluid from the GRS and CLV feeding systems appear to be more similar to each other (which is to be expected given the similarities of the diets), there is much clearer separation between the TMR and pasture-based systems. There are multiple rumen fluid metabolites that contribute to the separation between treatments in the PLS-DA model (*p* < 0.001 over 2000 permutations). The metabolites with the largest contribution in distinguishing the feeding systems are shown in the variable importance plot (Figure 4). The TMR and CLV rumen fluid had a significantly higher acetic acid concentration than GRS. Among other volatile fatty acids (VFAs) detected in the rumen fluid, CLV feeding was demonstrated to produce significantly higher concentrations of isovaleric acid and isobutyric acid. Volatile fatty acids are produced in the rumen as end products of microbial fermentation of proteins and carbohydrates, which represent a major source of energy for dairy cows [28]. Overall, increased VFA levels are indicative of increased rumen fermentation levels which could be as a result of increased dry matter intake (DMI) of TMR (~20 kg DM/cow/day for TMR vs. ~18 kg DM/cow/day for pastures), while Lüscher et al. [29] also discuss how organic matter digestibility, net energy concentration, and supply of metabolizable protein are generally higher for white clover than grasses. In particular, increased acetic acid could be as a result of increased cellulose consumption, which is fermented in the rumen to VFA, particularly acetic acid [30]. Among the amino acids, l-alanine and glycine were significantly higher in the rumen of cows fed CLV than both GRS and TMR diets. The CLV-fed cows’ rumen also had significantly higher concentrations of tyrosine than GRS-fed cows. The rumen of TMR and CLV-fed cows had significantly higher concentrations of l-glutamic acid and aspartate. Increased levels of amino acids could potentially be attributed to increased crude protein content of the feed providing a proteinaceous substrate for microbial degradation (see Table 2). Leucine and valine were significantly higher in the rumen of CLV-fed cows than that of GRS. Increased levels of these amino acids could in turn contribute to increased presence of isoacids, isobutyric, and isovaleric acid [28,31]. This increase in the presence of isoacids is of interest as Andries et al. [31] noted that, from an extensive review of cattle experiments, it appeared that, in lactating cows, a nutritional supplement of isoacids may have a positive influence on milk production. Indeed, the CLV system has been shown to produce significantly higher milk yields than that of GRS [5]. Each feeding system had a significant effect on the concentrations of dimethyl sulfone, with CLV > GRS > TMR (Figure 3). This compound was also the major compound responsible for the separation between the diets seen by PLS-DA (Figure 4). Dimethyl sulphone in the rumen is produced by the catabolism of sulfur amino acids, in particular methionine, which is hydrolysed to dimethyl sulphide [32] and subsequently oxidised to dimethyl sulfone. Methionine has been previously reported to be in higher concentrations in pastures as opposed to silage and hay diets [33]. As such, increased levels of dimethyl sulfone from pasture feeding could be related to increased levels of dietary crude protein as dimethyl sulfone is highest in the ruminal fluid of CLV-fed cows.

The TMR derived rumen fluid had significantly higher concentrations of 3-phenylpropionate compared to that of the rumen from pasture fed cows, while CLV feeding resulted in significantly higher concentrations of phenylacetate in the rumen compared to both TMR and GRS. These compounds have been identified previously as important aromatic acids in ruminal fluid [10]. They are generated through the hydrogenation of plant phenolic compounds such as *p*-coumaric, ferulic, and caffeic acid by ruminal micro-organisms [34]. A concomitant study analysing the milk from these diets identified, through headspace analysis of volatile compounds of the feeds, that TMR had significantly higher content of phenols than that of pasture feeds [35]. This could be attributed to the diversity of the ration mix and inclusion of grass and maize silage; as Martin [36] reported, phenols can be produced during the ensiling process. Ametaj et al. [9] also stated that increased phenyl acetate present in the rumen fluid could be a result of deamination of aromatic amino acids such as tyrosine which, as mentioned, was significantly higher in the rumen of CLV-fed cows.

The TMR feeding system resulted in significantly higher concentrations of the disaccharide d-maltose, compared to pasture feeding systems (Table 1). This is to be expected considering the much higher starch content of the TMR diet (Table 3 and Table 4). Similar increasing trends in the concentration of d-maltose with increasing proportions of cereal grain in the diet have been previously reported [6,9,10]. Pasture feeding resulted in significantly higher content of uracil in the rumen than that of TMR. Uracil has been reported previously in the rumen fluid metabolome by Ametaj et al. [9], and recently by Zhang et al. [37]. Both studies reported contrary results to this experiment, with an increase in rumen uracil concentrations from high concentrate diets. Uracil, alanine, and hypoxanthine in the rumen have been reported as products of bacterial degradation [38]. The CLV-fed cows rumen had significantly higher concentrations of acetone and isopropanol than those of TMR and GRS. Bruss and Lopez [39] concluded through the use of in vitro experiments that rumen microbial metabolism of acetone is the likely source of plasma isopropanol. Martin et al. [40] recently demonstrated that isopropanol can also be produced through rumen hydrolysis of the methionine analogues, butanoic acid and its isopropyl ester. The ruminal fluid from CLV-fed cows had significantly higher concentrations of nicotinate than that of TMR and GRS-fed cows. Nicotinate has been reported in the rumen fluid metabolome previously [9,10], however, in these studies its concentration was not affected by diet but its presence was attributed to the ability of several bacterial species identified in rumen contents to synthesize nicotinate.

*p*-Cresol is a metabolite that has received much attention in recent years as a result of its effects on the sensory quality of pasture derived milk and milk products. Interestingly, the rumen of CLV-fed cows had significantly higher concentrations of *p*-cresol than that of TMR and GRS. Although not significantly different (*p* > 0.05), average *p*-cresol content of GRS was also considerably higher than that of TMR (65.95 μM vs. 58.38 μM respectively). The deviation of *p*-cresol concentrations throughout the study appeared to be considerably higher in pasture cows than that of TMR which is likely in response to changing pasture quality and intake over the season. Martin [36] concluded that *p*-cresol is a rumen metabolite that is produced through the deamination and decarboxylation reactions associated with the degradation of tryptophan and tyrosine [41]. Increased *p*-cresol could also be as a result of *β*-carotene degradation [42]. *β*-carotene is naturally higher in fresh pastures as the ensiling process and processing of feeds for concentrates typically depletes or destroys many carotenoids, and as a result most concentrates fed to cows on a TMR system are naturally low in *β*-carotene [43]. As such, *β*-carotene has been suggested as a biomarker of fresh pasture feeding in dairy products [44]. Finally, elevated levels of *p*-cresol in CLV could be as a result of formononetin, a constituent of clover species, which has been shown to be degraded in the rumen to produce *p*-cresol [45]. Overall, these alterations would suggest that the dietary regimen is altering the metabolome, potentially through alterations in microbiome functionality (rather than overall gross structure).

Total mixed ration feeding resulted in significantly higher choline content in ruminal fluid than pasture feeding. Choline is regarded as an important compound for cow health status and has been suggested to impact milk production [46]. Choline can be acquired in two major forms; through diet, although in ruminants dietary choline is extensively degraded in the rumen [47], and via endogenous synthesis by the phosphatidylethanolamine *N*-methyltransferase (PEMT) pathway, which represents an important source of choline [48]. Choline is an important compound as it can act as a precursor for several metabolites such as acetylcholine (a neurotransmitter) and betaine (a source of labile methyl groups), and is required to make essential membrane phospholipids [49]. Phosphatidylcholine, in particular, is produced via the cytidine diphosphate (CDP) choline pathway [48] and is important for the removal of triacylglycerol from the liver. As such, an absence of choline can result in fatty liver degeneration and choline is considered a lipotropic factor [50]. Higher choline availability achieved through feeding strategies utilizing rumen protected choline has been shown to have a favourable effect on milk production. This could be attributed to subsequent increased availability of methionine for milk synthesis, enhanced glycogenesis in the liver, and general health improvement of the cows [50,51].

The CLV feeding resulted in significantly higher ruminal content of formate than GRS, while TMR feeding resulted in significantly higher rumen concentrations of succinate than that of GRS feeding. Formate and succinate have been described as important fermentation products of pure cultures of rumen bacteria [52,53]. Succinate is an important metabolite in rumen fermentation, and is produced as a byproduct of carbohydrate fermentation. It is decarboxylated by a series of enzymes to form propionate. Propionate formation is essential to ruminants as a substrate for glucose synthesis [53]. Formate is metabolised rapidly in the rumen and can be an important source of hydrogen, which, when coupled with carbon dioxide, appear to be the chief substrates for methanogenisis [54]. Indeed, Lovley et al. [55] reported that methanogenic bacteria have the potential to directly metabolize formate in the rumen to produce methane. This could, in turn, be disadvantageous in comparison to GRS, with potentially increased production of methane adding to overall greenhouse gas emissions. Further work is, however, required to confirm this.

Although not the main focus of this study, a change in the rumen metabolome was observed between different stages of lactation (Supplementary Materials Table S1). The PLS-DA (Supplementary Materials Figure S2) shows that, while the rumen metabolomes of mid and late lactation are very similar and cluster together, early lactation appears to be more distinguished and separates from the other stages of the lactation cycle. Alterations of the rumen metabolome occurring throughout lactation are to be expected as a result of the changing quality and availability of pastures through the seasons (Table 2 and Table 3) and energy requirements of the cow throughout lactation. There was a Diet*SOL (stage of lactation) interaction (*p* < 0.01) also seen for several metabolites including phenylacetate, isovaleric acid, isopropanol, isobutyric acid, *p*-cresol, 3-phenylpropionate, succinate and aspartate. Such changes are likely as a result of changing intake and pasture quality during peak milk production early-lactation period. However, major differences in the early lactation period observed in PLS-DA (Supplementary Materials Figure S2 could also be as a result of negative energy balance experienced in cows at the onset and during early lactation. In early lactation, dietary intake is often unable to meet the demands of high milk production. The cow therefore enters a period of negative energy balance, causing mobilization of body reserves to balance the deficit between food energy intake and milk energy production, which can result in alterations to metabolism [56].

### 2.3. Milk Metabolome

**Figure 5:**
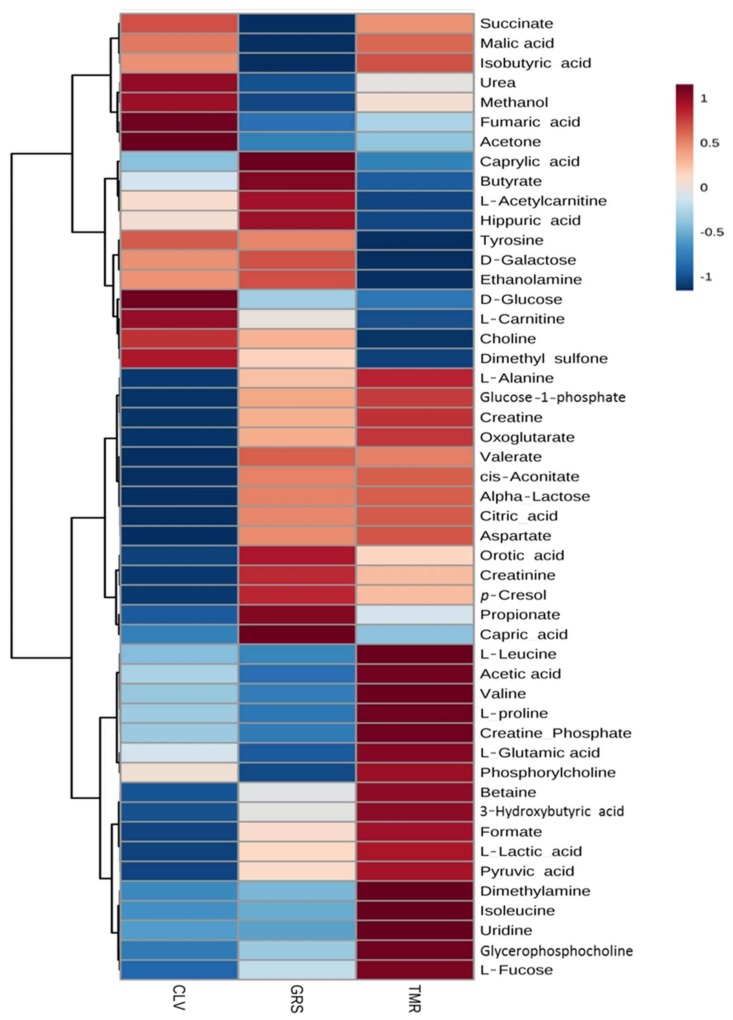
Hierarchical clustering analysis (heatmap) of average raw milk metabolites from mid-lactation cows fed diets consisting of total mixed ration (TMR), perennial ryegrass (GRS), or perennial ryegrass and white clover (CLV), as determined by ^1^H-NMR. The degree of positive and negative correlation of metabolite to dietary system is indicated by +1 (red) to −1 (blue).

**Figure 6:**
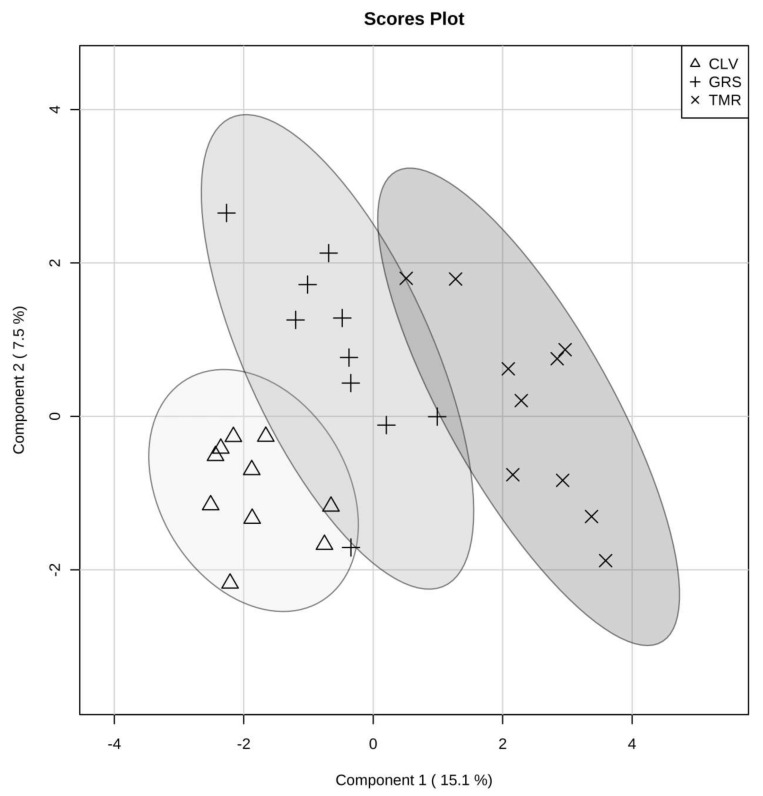
Partial least square discriminant analysis (PLS-DA) score plot of mid-lactation raw milk metabolome of cows fed diets consisting of total mixed ration (TMR), perennial ryegrass (GRS), or perennial ryegrass and white clover (CLV), as determined by ^1^H-NMR. The shaded ellipses represent the 95% confidence interval estimated from the score.

^1^H-NMR analysis of mid-lactation milk samples identified 49 metabolites present in each of the samples, the majority of which were also found in the rumen fluid portion. Table 5 shows the average concentration of each of the metabolites from milk from each of the feeding systems. The feeding system was shown to have a significant effect on the milk metabolome (Figure 5). Such differences are clearly visible using PLS-DA (Figure 6, *p* < 0.005 on 2000 permutations). These results demonstrated that it is possible to distinguish milk from each of the feeding systems from each other by their metabolomes using ^1^H-NMR-based metabolomics (Figure 5 and Figure 6). Similar to the ruminal fluid data, the milk data from GRS and CLV-fed cows appear to be more similar to each other although with a more obvious separation than that observed in the rumen (Supplementary Materials Figure S1). In addition, there is a much clearer separation between the TMR and pasture-based systems (Figure 6). The PLS-DA also allowed the identification of the metabolites that were most important for the observed separation in milk samples, as shown in Supplementary Materials Figure S3, variables with VIP values > 1. ANOVA analysis and the multiple comparison tests of milk metabolites showed similar patterns to the rumen-fluid; CLV derived milk had significantly higher concentrations of acetone, while tyrosine concentrations were also greater in pasture derived milk than that of TMR. While following a similar trend to that of rumen acetone levels, the significantly increased acetone in CLV derived milk is still poorly understood. Faulkner et al. [35] also reported finding acetone as a volatile in the milk and feed samples of these diets, where acetone levels were significantly higher in the CLV feed samples (peak area (PA) 6.88 × 10^7^) than GRS (PA 2.62 × 10^5^) and TMR (PA 1.72 × 10^7^) samples. Contarini et al. [57] also highlighted that milk acetone can originate directly from the feed. Heuer et al. [58] discussed a potential relationship between high milk producing cows and acetone, whereby high milk producing cows have a greater capacity for the clearance of acetyl-CoA via ketogenesis, leading to increased synthesis of acetone and its excretion in milk. Although no ketosis was observed in the cows in this study, acetone concentration in milk has previously been correlated with subclinical and clinical ketosis in dairy cows; and as such acetone in milk at levels of 0.7 to 1.4 mmol has been suggested as a warning of hyperketonaemia in cows [59]. Each diet had a significant effect on the concentration of dimethyl sulfone with CLV > GRS > TMR. Dimethyl sulfone has previously been reported at significantly increased concentrations in milk derived from pasture [33,60]. The TMR derived milk had significantly higher concentrations of dimethylamine than both pasture derived milks. Saleem et al. [10] reported the presence of dimethylamine in the rumen of cows fed increasing proportions of grain, and the presence of biogenic amines is related to dietary source and alteration to the rumen microbiota. Each feeding system also had a significant effect on the concentration of urea in milk with CLV > TMR > GRS. Urea is a typical constituent of milk and contributes a major portion of the non-protein nitrogen fraction of milk. Indeed, levels of urea align with the non-protein nitrogen content of milk from these diets [5]. Urea is the metabolic end product of protein catabolism in the body [61]. As such, the concentration of milk urea nitrogen is influenced by dietary crude protein intake and digestibility. Harris et al. [62] also reported that increased clover proportions in the diet resulted in increased urea concentrations. Crude protein is digested in the rumen producing ammonia, part of which is taken up by the blood stream and transported to the liver where it is converted to urea, which is then diffused into the milk and blood [63]. Indeed, milk urea levels are correlated with blood urea levels. Huhtanen et al. [64] demonstrated that an increase in milk urea nitrogen is negatively associated with efficiency of nitrogen utilisation and positively associated with urea excretion. Reduced urea excretion would be advantageous in reducing the agricultural contribution to environmental pollution [63]. Furthermore, increased levels of urea will also contribute to increased milk non-protein nitrogen content, which would be disadvantageous to dairy manufacturers. This is because current milk payment schemes, in certain markets, are based on milk crude protein as opposed to true protein content. Therefore, increased milk urea potentially results in reduced product yields. Pasture feeding resulted in milk with significantly higher hippuric acid content than that of TMR. Likewise, GRS milk hippuric acid was significantly higher than CLV. Hippuric acid has been identified as a constituent of the non-protein nitrogen fraction of milk [65]. The presence of hippuric acid in milk has been attributed to the presence of caffeoylquinic compounds in forages, and increased levels of hippuric acid in milk from pasture-based feeding systems has previously been reported in milk from cows and goats [66,67]. Boudonck et al. [68], using GC-MS metabolomics, also reported significantly higher content of hippuric acid in pasture versus conventional milk. Increased levels of hippuric acid from pasture-based feeding are in agreement with the results of Carpio et al. [69], who, using goats milk, suggested hippuric acid as a biomarker of feeding systems, where increased levels of hippuric acid represents a diet based mainly or exclusively on grazing pastures.

## 3. Materials and Methods

### 3.1. Experimental Design and Sample Collection

Three feeding systems were compared over a full lactation at the Teagasc, Animal and Grassland Research and Innovation Centre, Moorepark, Fermoy, Co. Cork, Ireland. Herd 1 was housed indoors and fed a total mixed ration diet (TMR), herd 2 was maintained outdoors on perennial ryegrass only pasture (GRS), while herd 3 was also maintained outdoors on a perennial ryegrass/white clover pasture (CLV). The TMR group were offered on a DM basis, 7.15 kg grass silage, 7.15 kg maize silage, and 8.30 kg concentrates (see Table 3 and Table 4). Cows on pasture received a mineral supplement in the form of a liquid mineral preparation injected into the water supply (Terra Liquid Minerals, Moone Lodge, Moone, Athy, Co., Kildare, Ireland), giving a mean intake (mg/cow per day) of Na, Mg, Zn, Cu, Se, and Co of 5.0, 1.2, 219, 106, 3.8, and 3.0, respectively. The concentrate portion of the TMR feed was supplemented with a commercial mineral balancer, Dairy Hi-Phos (McDonnell Bros. Agricultural Suppliers Ltd., Fermoy, Co., Cork, Ireland) to give added Ca, Na, P, Zn, Cu, Mn, I, Co, and Se of 3340, 2000, 1200, 140, 100, 70, 10, 2, and 0.8 mg/kg, respectively [70].

Cows within the TMR system were fed at 08:30 h daily into electronically controlled Griffith Elder Mealmaster individual feed bins (Griffith Elder and Company Ltd., Suffolk, UK). Feed was available ad-libitum and cows consumed on average across the year 19.50 kg DM/cow/day. Pasture-based cows consumed ~18 kg DM/day, measured by pre- and post-grazing sward heights daily using the rising plate meter (Jenquip, Fielding, New Zealand), while pre-grazing herbage mass was measured with an Etesia mower (Etesia UK Ltd., Warwick, UK). The CLV sward contained ~20% white clover and was measured according to Egan et al. [71]. Compositional analysis of GRS and CLV swards are shown in Table 2.

Rumen sampling took place at 07:00 each morning. Nine rumen cannulated, spring calving Friesian cows were allocated to three groups (*n* = 3). Prior to the experimental period, cannulated cows were on a perennial ryegrass diet, three cows were randomly assigned to each diet for a 16 day period; the first two weeks was an acclimatization period and rumen samples were collected on the morning of day 15 and 16. The cows were then rotated to a different feeding system and the two-week acclimatization process was repeated. This sampling process was performed in each stage of lactation (early, mid and late). Rumen samples were collected in a similar manner to that described by O’Connor et al. [72]. Briefly, rumen contents were collected and subsequently squeezed and filtered through three layers of synthetic cheese cloth. The rumen fluid portion which permeated the cheese cloth, and the retained solids portion were collected in sterile containers and stored at −80 °C prior to analysis.

For milk collection and analysis, fifty-four spring calving Friesian cows were allocated to three groups (*n* = 18) at the Teagasc, Animal and Grassland Research and Innovation Centre, Moorepark, Fermoy, Co. Cork, Ireland in February 2016. Groups were randomized based on milk yield, milk solids yield, calving date and lactation number. Group 1 was housed indoors and fed a TMR diet, Group 2 was maintained outdoors on perennial ryegrass only pasture (GRS), while Group 3 was also maintained outdoors on a perennial ryegrass/white clover pasture (CLV) [5]. Cows remained on their diet treatment for the entire lactation period.

Cows were milked at 07:00 and 15:30 daily. Milk from ten non-cannulated individual cows in each of the groups was collected in July 2016, when the cows were in the mid stage of lactation. On the first day of collection, 10 cows from each group were randomly selected and the same 10 cows were used for subsequent milk sampling. Milk from cows in each of the feeding systems was collected during the morning and evening milkings and individual cow samples for each day were combined 1:1 (*v*/*v*) in a 50 mL falcon tube which was then vortexed, and stored at −80 °C. Milk collections were performed from each feeding system in triplicate one day apart (i.e., Monday, Wednesday, and Friday) to provide a comprehensive sample set of milk at that particular time of lactation.

### 3.2. Feed Compositional Analysis

Feed samples were collected throughout lactation from the paddocks at the time of grazing. Grass silage samples were collected weekly. Samples were dried at 60 °C for 48 h, milled, and stored prior to analysis. Samples were analysed using near infrared reflectance spectroscopy using a FOSS 6500 (FOSS Ireland Ltd., Dublin, Ireland). The UFL, PDIA, PDIE, PDIN have been calculated according to the INRA feeding system equations [73]. Analysis of maize silage was carried out by FBA Laboratories Ltd. (Waterford, Ireland).

### 3.3. Ethical Approval

Teagasc has both an animal welfare body (AWB) and animal ethics committee. The AWB is a legal requirement of Article 26 of Directive 2010/63/EU and Regulation 50 of S.I. No. 543 of 2012. The Health Products Regulatory Authority (HPRA) provides project authorization and the HPRA License number for this project is AE19132/P019.

### 3.4. NMR Sample Preparation

^1^H-NMR analysis of ruminal fluids and raw milk samples was performed at The Metabolomics Innovation Centre (TMIC), University of Alberta, Edmonton, AB, Canada. Sample preparation for NMR analysis was performed using a similar method as described by Saleem et al. [6,10]. Briefly, 3 kDa filters (Amicon Micoron YM-3; Sigma-Aldrich, St. Louis, MO, USA) were washed five times using 500 µL HPLC-graded water and centrifuged at 10,000 RPM for 10 min. Samples were thawed at 4 °C overnight the day before analysis; once thawed, rumen fluid samples were vortexed and then centrifuged at 3000 RPM for 5 min to sediment any particulate matter. 400 µL of sample fluid was filtered through washed 3 kDa filters at 11,000 RPM for 35 mins at 4 °C. 200 µL of filtrate was then added to 50 µL standard NMR buffer solution (5 mM DSS (disodium-2, 2-dimethyl-2-silapentane-5-sulphonate)), and 0.1% NaN_3_ in H_2_O (Sigma–Aldrich, Mississauga, ON, Canada) and transferred to standard NMR tubes. All ^1^H-NMR spectra were collected on a 700 MHz Bruker NMR spectrometer equipped with a 5 mm cryoprobe. ^1^H-NMR spectra were acquired at 25 °C using the first transient of the noesy-presaturation pulse sequence, which was chosen for its high degree of quantitative accuracy [74]. Spectra were collected with 128 transients using a 4 s acquisition time, a 2 s relaxation delay, and a 0.5 s mixing time.

### 3.5. NMR Compound Identification and Quantification

Prior to spectral analysis, all free induction decays (FIDs) were zero-filled to 64 k data points and a line broadening of 0.5 Hz was applied. The methyl singlet of the added DSS served as an internal standard for chemical shift referencing (set to 0 ppm) and for quantification. The resulting rumen ^1^H-NMR spectra were processed and analysed using BAYESIL (http://www.bayesil.ca), a fully-automated NMR spectral profiling program. Milk ^1^H-NMR spectra were processed and analysed using the Chenomx NMR suite (v 8.1, Chenomx Inc., Edmonton, AB, Canada). Each spectrum was processed and analysed by at least two experienced NMR spectroscopists to minimize compound mis-identification and mis-quantification.

### 3.6. DNA Extraction and MiSeq Sequencing

All rumen fluid and solid samples were individually ground to a fine powder under liquid nitrogen using a mortar and pestle. DNA was extracted using repeated bead beating plus column method according to Yu and Morrison [75].

The V3–V4 regions of the 16S rRNA gene were amplified and adapter sequences chosen according to the 16S metagenomic sequencing library protocol for the Illumina MiSeq using the following 16S primer pair Forward—(5′CGTCGGCAGCGTCAGATGTGTATAAGAGACAGCCTACGGGNGGCWGCAG) and Reverse—(5′GTCTCGTGGGCTCGGAGATGTGTATAAGAGACAGGACTACHVGGGTATCTAACC).

### 3.7. Bioinformatic (DNA) Analysis

Libraries were sequenced on a MiSeq sequencer (Illumina) using 2 × 250 bp chemistry. The 64-bit versions of USEARCH 9.2 [76] and mothur [77] was used in combination with several in-house programs for bioinformatical analysis of the sequencing data. Following tag identification and trimming, all sequences from all samples were pooled. Paired end reads were merged, requiring at least 10 bp overlap and a merged read length of between 300 and 500 bp. Sequences with ambiguous bases, without perfect match with the primers, or homopolymer length greater than 8 were discarded and primer sequences trimmed. Reads were quality filtered, discarding reads with more than 1 expected error and sequences strictly dereplicated, discarding clusters smaller than 5.

Sequences were clustered at 97% sequence similarity, using the most abundant strictly dereplicated reads as centroids and discarding suspected chimeras based on internal comparison. Taxonomic assignment of OTUs was done using the method by Wang et al. [78] with mothur’s PDS version of the RDP training database v14. Following this, samples were rarified to the lowest sequence number found in a sample ≥1000 (after in silico removal of contaminating OTUs).

### 3.8. Statistical Analysis

The rumen and milk samples were analysed separately. For the rumen samples, univariate statistical analysis was performed to discover potential differences between the feeding systems. General linear models (GLM) were built for each metabolite to determine whether the means of the three feeding systems (TMR, GRS, and CLV) differ. The stage of lactation (early, mid, and late) and the interaction with the diet were also included in the model. Data was log (base 2) transformed in order to reduce potential influential points and reduce the skew of the data. A paired t-test was used to examine the differences between rumen liquid and rumen solid portions, with an adjusted p-value of < 0.05 considered significant, *t*-test analysis was carried out using R.

For the milk study, a total of 30 cows were split randomly in three balanced but different dietary treatments (TMR, GRS, and CLV). Metabolites were analysed in the mid-lactation period for three consecutive days. For each cow the values of each repetition were averaged by the mean. One-way analysis of the variance (ANOVA) was performed by building a regression model for each metabolite to compare the mean values between the three different groups. Prior to the analysis, data was also log transformed. In both studies the type I error, due to multiple testing, was controlled by using the Benjamini and Hochberg false discovery rate (FDR) procedure. Two-sided *p*-values < 0.05 of the corrected *p*-values was used as the threshold to refuse the null hypothesis that the means of all the groups did not differ. Tukey’s Honestly Significant Difference (HSD) test was used as a post-hoc analysis to find which treatments were significantly different from each other (*p* < 0.05) among the significant metabolites.

For both rumen and milk studies, multivariate analyses were performed. Unsupervised hierarchical clustering analysis (HCA) was done to observe patterns in the data, and is shown as a heatmap. A supervised multivariate model was built using partial least squared discriminant analysis (PLS-DA). In order to validate the model, a permutation test with 2000 repetitions was performed to check that the model differed from a random model. Also the R^2^ and Q^2^ parameters were obtained to assess the performance of the model using a 10 fold cross-validation approach as well as to estimate the number of components to analyse. The variable importance plot (VIP) shows which variables have a larger influence to the latent variables of the built model. Metaboanalyst [79] software and in-house R (R Core Team, version 3.4.2, 2016) scripts were used to perform the multivariate statistical analysis and produce the figures.

## 4. Conclusions

In line with previous studies, the rumen microbiota was found to be remarkably complex and stable over time. While no significant differences in the rumen microbiota composition between cows exposed to different diets was found, the type of feeding system used to nourish dairy cows was shown to have a significant effect on their rumen and milk metabolome. Among other compounds, increased concentrations of VFAs (i.e., acetic acid), an important source of energy for the cow, were detected in the rumen of cows associated with TMR and CLV feeding systems. When analysing milk, we found that CLV and TMR feeding systems resulted in increased concentrations of milk urea. Urea is indicative of the nitrogen metabolism efficiency in the rumen, and can negatively affect milk protein quality with increased non-protein nitrogen content. Our results suggest that NMR-based metabolomics could be a useful tool for milk verification purposes in the future as “pasture” milk and dairy products become more popular with consumers. Further work analysing variations in the dairy cow’s diet with respect to level of pasture and concentrate supplementation will offer important insights into the robustness of the methodology. Finally, this study also demonstrates that short term changes in diet do not affect the gross overall microbiome structure of the rumen. Rather, these short-term dietary changes seem to affect the functionality of the community, which obviously responds in a far shorter time period to cause changes in the metabolome.

## Figures and Tables

**Table 1:** Average concentrations (Mean ± standard deviation) of rumen metabolites (μM) measured in the rumen of lactating dairy cows fed diets consisting of total mixed ration (TMR), perennial ryegrass (GRS), or perennial ryegrass and white clover (CLV), as determined by ^1^H-NMR. Adjusted *p*-values from the ANOVA test for the feed system, lactation period, and their interaction.

Metabolite (μM)	Diet			*p*-Value		
	TMR	GRS	CLV	Diet	Time	Diet × Time
2-hydroxyisovalerate	7.06 (±6.35)	6.17 (±4.99)	7.49 (±5.32)	0.78	0.81	0.21
3-Hydroxybutyric acid	12.78 (±14.29)	8.68 (±5.09)	12.63 (±13.27)	0.51	0.13	0.28
3-Hydroxyphenylacetic acid	26.71 (±9.76)	22.41 (±9.00)	22.85 (±7.60)	0.25	<0.01	0.16
3-Phenylpropionate	745.91 (±112.81) ^b^	632.94 (±170.49) ^a^	634.19 (±124.84) ^a^	<0.01	<0.01	<0.01
4-Aminobutyrate	36.39 (±18.73)	52.15 (±35.68)	48.77 (±20.07)	0.36	0.26	0.86
Acetic acid	55,295.87 (±7149.21) ^b^	54,836.35 (±6828.43) ^a^	58,322.59 (±3754.77) ^b^	0.01	<0.01	0.03
Acetoin	25.41 (±9.68)	21.36 (±6.28)	25.29 (±8.02)	0.26	0.1	0.68
Acetone	8.67 (±3.16) ^a^	8.53 (±2.01) ^a^	12.03 (±6.44) ^b^	0.01	<0.01	0.07
Adenine	26.43 (±7.53)	26.12 (±12.30)	30.54 (±22.58)	0.51	<0.01	0.22
Adenosine	4.55 (±3.34)	6.78 (±5.51)	5.08 (±4.26)	0.35	0.16	0.49
Aspartate	153.24 (±54.47) ^b^	115.83 (±45.41) ^a^	148.01 (±79.63) ^b^	0.03	<0.01	<0.01
Benzoic acid	23.88 (±4.87)	26.31 (±7.47)	27.46 (±5.34)	0.02 ^d^	<0.01	0.04
Beta Alanine	18.43 (±14.98)	18.24 (±16.25)	17.44 (±14.00)	0.94	<0.01	0.01
Betaine	6.91 (±10.81)	5.08 (±5.66)	3.80 (±1.87)	0.51	0.36	0.79
Butyrate	12,585.29 (±2066.81)	14,245.39 (±2839.45)	14,728.79 (±2514.33)	0.02 ^d^	0.05	0.21
Cadaverine	82.27 (±39.12)	96.51 (±76.25)	107.09 (±27.17)	0.43	0.97	0.16
Choline	25.89 (±14.19) ^b^	14.68 (±8.23) ^a^	12.59 (±8.65) ^a^	<0.01	0.19	0.03
*cis*-Aconitate	5.58 (±3.40)	8.46 (±9.56)	7.59 (±5.55)	0.50	0.30	0.44
Citric acid	7.46 (±6.93)	7.53 (±4.36)	6.62 (±2.72)	0.83	0.02	0.67
Creatine	8.69 (±5.98)	6.93 (±5.02)	6.93 (±3.56)	0.51	0.71	0.96
d-Glucose	520.63 (±287.13)	544.86 (±383.52)	602.03 (±536.85)	0.63	<0.01	0.03
d-Maltose	71.21 (±66.10) ^b^	34.40 (±27.63) ^a^	33.35 (±26.23) ^a^	0.02	<0.01	0.18
Dimethyl sulfone	3.83 (±2.43) ^a^	16.81 (±7.70) ^b^	33.38 (±12.49) ^c^	<0.01	0.03	0.21
Dimethylamine	4.25 (±7.97)	2.31 (±1.32)	4.80 (±9.51)	0.64	0.26	0.69
Dimethylglycine	14.28 (±21.99)	4.55 (±4.93)	7.24 (±13.14)	0.31	0.81	0.79
Ethanol	25.63 (±22.49)	59.29 (±109.16)	31.70 (±20.26)	0.37	0.66	0.28
Ethanolamine	29.38 (±13.94)	28.26 (±13.02)	29.48 (±19.30)	0.94	<0.01	0.44
Formate	118.49 (±4.16) ^a,b^	114.91 (±5.35) ^a^	118.09 (±2.89) ^b^	0.01	0.01	0.06
Glycerol	254.92 (±55.91) ^b^	242.33 (±34.69) ^a^	248.91 (±48.42) ^a,b^	0.51 ^d^	<0.01	0.28
Glycine	121.49 (±37.66) ^a^	119.16 (±40.23) ^a^	158.48 (±60.01) ^b^	0.01	<0.01	0.03
Hypoxanthine	171.16 (±32.82) ^b^	159.21 (±56.02) ^a^	179.41 (±70.06) ^b^	0.27 ^d^	<0.01	0.03
Inosine	11.53 (±6.29) ^a^	27.24 (±26.18) ^b^	22.14 (±18.14) ^b^	0.01	<0.01	0.03
Isobutyric acid	796.76 (±88.10) ^a^	811.95 (±103.96) ^a^	997.57 (±191.24) ^b^	<0.01	<0.01	<0.01
Isoleucine	84.44 (±23.65) ^a,b^	83.46 (±35.14) ^a^	105.76 (±38.25) ^b^	0.07 ^d^	<0.01	0.27
Isopropanol	18.16 (±3.86) ^a^	22.25 (±6.37) ^a^	42.18 (±27.01) ^b^	<0.01	<0.01	<0.01
Isovaleric acid	683.44 (±112.48) ^a^	701.31 (±135.43) ^a^	951.09 (±283.61) ^b^	<0.01	<0.01	<0.01
l-Alanine	180.40 (±45.02) ^a^	189.58 (±55.46) ^a^	238.88 (±76.37) ^b^	<0.01	<0.01	0.04
l-Glutamic acid	293.98 (±71.48) ^b^	252.06 (±72.63) ^a^	350.58 (±97.44) ^b^	<0.01	0.02	0.06
l-Histidine	32.96 (±11.46)	36.68 (±14.08)	34.49 (±15.58)	0.65	<0.01	0.32
l-Lactic acid	30.66 (±22.16)	29.41 (±44.10)	27.49 (±18.07)	0.94	0.06	0.54
l-Leucine	89.73 (±19.51) ^a,b^	93.85 (±31.78) ^a^	119.58 (±38.95) ^b^	0.01	<0.01	0.21
l-Lysine	149.54 (±64.36)	173.82 (±77.85)	205.94 (±123.11)	0.14	<0.01	0.45
l-Phenylalanine	51.48 (±13.84)	50.64 (±19.03)	57.76 (±22.60)	0.32	<0.01	0.17
l-Proline	84.33 (±34.94)	80.50 (±37.46)	108.33 (±63.16)	0.09	<0.01	0.09
l-Threonine	108.67 (±41.15) ^b^	85.44 (±32.09) ^a^	106.61 (±42.64) ^a,b^	0.08 ^d^	<0.01	0.04
Methanol	13.74 (±14.69)	9.69 (±1.28)	11.44 (±3.02)	0.51	0.81	0.44
Methionine	33.63 (±9.78)	35.13 (±13.72)	40.76 (±12.37)	0.26	0.01	0.34
Methylamine	4.64 (±11.01)	11.39 (±16.79)	16.25 (±33.42)	0.25	0.15	0.04
Nicotinate	25.77 (±7.67) ^a^	28.56 (±9.80) ^a^	35.93 (±9.57) ^b^	<0.01	<0.01	0.03
*o*-Hydroxyphenylacetic acid	17.38 (±3.47) ^c^	13.08 (±3.78) ^a^	15.66 (±5.49) ^b^	<0.01	<0.01	0.03
*p*-Cresol	58.38 (±15.58) ^a^	65.95 (±24.67) ^a^	85.18 (±38.62) ^b^	<0.01	<0.01	<0.01
*p*-Hydroxyphenylacetic acid	17.86 (±4.95)	14.62 (±3.72)	13.77 (±4.27)	0.07	0.87	0.72
Phenylacetate	199.58 (±56.74) ^a^	262.11 (±109.84) ^a^	381.02 (±191.25) ^b^	<0.01	<0.01	<0.01
Propionate	17,119.26 (±3923.21)	18,084.74 (±3647.63)	18,140.78 (±2724.51)	0.43	0.01	0.04
Putrescine	58.93 (±21.87)	56.48 (±55.10)	45.56 (±16.64)	0.51	0.02	0.56
Succinate	123.57 (±122.66) ^b^	74.56 (±51.26) ^a^	90.93 (±54.20) ^a,b^	0.01	<0.01	<0.01
Trimethylamine	5.80 (±14.48)	1.85 (±1.38)	5.40 (±9.03)	0.51	0.37	0.13
Tryptophan	7.17 (±1.65)	7.39 (±3.02)	8.19 (±3.41)	0.36	<0.01	0.11
Tyrosine	44.27 (±12.59) ^a,b^	43.55 (±16.83) ^a^	56.05 (±20.71) ^b^	0.03	<0.01	0.21
Uracil	200.49 (±91.29) ^a^	264.18 (±106.92) ^b^	297.61 (±107.14) ^b^	<0.01	<0.01	0.55
Uridine	7.40 (±8.28)	7.49 (±5.34)	8.81 (±8.03)	0.83	0.42	0.28
Valerate	1030.42 (±266.50)	1256.89 (±602.06)	1328.14 (±439.27)	0.08	0.01	0.04
Valine	93.59 (±23.59) ^a,b^	90.46 (±35.70) ^a^	128.79 (±65.36) ^b^	0.01	<0.01	0.11

^a,b,c^ indicates significant differences from Tukey Honestly Significant Difference (HSD) pairwise test statistical between treatments; ^d^ Indicates attributes where significant differences were not found for both statistical analysis by ANOVA and Tukey Honestly Significant Difference pairwise comparison.

**Table 2:** Chemical composition and nutritional content (mean ± SD) of pasture system forages (GRS and CLV), collected weekly throughout lactation, analysed by near-infrared spectroscopy.

	Early Lactation		Mid Lactation		Late Lactation	
	GRS	CLV	GRS	CLV	GRS	CLV
OM (g/kg of DM)	881.2 ± 22.3	883.7 ± 32.4	864.1 ± 42.8	867.2 ± 32.8	807.5 ± 49.9	814.8 ± 28.2
CP (g/kg of DM)	218.4 ± 30.7	228.1 ± 19.3	216.9 ± 30.0	239.9 ± 28	266.5 ± 17.2	279.7 ± 25.2
ADF (g/kg of DM)	284.6 ± 18.4	283.0 ± 23.1	294.1 ± 17.1	298.4 ± 21.1	321.7 ± 24.7	314.3 ± 23.2
NDF (g/kg of DM)	378.2 ± 21.5	350.4 ± 27.5	386.1 ± 31.0	366.3 ± 31.2	429.5 ± 35.9	385.4 ± 22.0
Ash (g/kg of DM)	61.8 ± 10.1	60.4 ± 9.2	68.7 ± 16.9	61.8 ± 11.4	75.1 ± 8.9	65.1 ± 10.5
UFL (/kg of DM)	0.96 ± 0.03	0.97 ± 0.04	0.94 ± 0.03	0.95 ± 0.04	0.91 ± 0.05	0.94 ± 0.04
PDIA (/kg of DM)	42.8 ± 4.0	44.0 ± 2.5	42.6 ± 3.9	45.6 ± 3.7	49.1 ± 2.5	51.1 ± 3.7
PDIE (/kg of DM)	101.2 ± 4.0	102.9 ± 1.9	99.7 ± 3.9	103.4 ± 3.5	104 ± 3.6	108.4 ± 3.2
PDIN (/kg of DM)	141.1 ± 20.6	147.4 ± 13.0	140.0 ± 20.0	155.4 ± 18.9	173.5 ± 11.9	182.8 ± 17.5

Note: OM = organic matter, CP = crude protein, UFL = unité fourragère lait; PDIA = sum of the feed protein ruminally undegraded and truly digested in the small intestine; PDIE = sum of PDIA and the microbial true protein that is truly digested in the small intestine (PDIM) when energy is limiting; PDIN = sum of PDIA and PDIM when nitrogen is limiting.

**Table 3:** Chemical composition (mean ± SD) and nutritional content of silages from TMR diet (grass silage and maize silage) collected weekly throughout lactation analysed by near-infrared spectroscopy.

	Early Lactation		Mid Lactation		Late Lactation	
	Grass Silage	Maize Silage	Grass Silage	Maize Silage	Grass Silage	Maize Silage
DM (g/kg of DM)	238.6 ± 12.1	279.5 ± 13.9	466.8 ± 83.3	280.0 ± 25.7	340.2 ± 155.6	238.7 ± 3.3
CP (g/kg of DM)	96.7 ± 6.4	66.3 ± 8.1	128.2 ± 10.5	56.7 ± 5.0	118.4 ± 7.8	64.7 ± 9.0
Starch (g/kg of DM)	NA	221.3 ± 14.9	NA	235.7 ± 33.7	NA	198.3 ± 6.3
ADF (g/kg of DM)	262.5 ± 12.2	NA	270.6 ± 6.1	NA	275.7 ± 17.7	NA
NDF (g/kg of DM)	386.5 ± 27.4	470.5 ± 32.2	407.3 ± 10.3	510.3 ± 9.0	409.6 ± 35.7	522.7 ± 4.7
ASH (g/kg of DM)	92.3 ± 5.9	27.0 ± 1.6	82.8 ± 2.5	28.3 ± 4.6	89.1 ± 4.6	29.7 ± 0.5
UFL (/kg of DM)	1.02 ± 0.03	0.90 ± 0.1	1.0 ± 0.02	0.90 ± 0.1	0.97 ± 0.05	0.9 ± 0.1
PDIA (/kg of DM)	17.0 ± 1.0	14.4 ± 1.8	31.0 ± 5.1	12.3 ± 1.1	24.6 ± 6.7	14.1 ± 2.0
PDIE (/kg of DM)	57.0 ± 4.1	64.5 ± 1.7	81.0 ± 8.2	60.9 ± 0.8	72.6 ± 7.2	62.5 ± 2.2
PDIN (/kg of DM)	70.6 ± 1.2	40.7 ± 5.0	77.5 ± 4.8	34.8 ± 3.1	70.6 ± 6.8	39.7 ± 5.5

Note: DM = Dry Matter, CP = Crude Protein, UFL = unité fourragère lait; PDIA = sum of the feed protein ruminally undegraded and truly digested in the small intestine; PDIE = sum of PDIA and the microbial true protein that is truly digested in the small intestine (PDIM) when energy is limiting; PDIN = sum of PDIA and PDIM when nitrogen is limiting. NA = not available.

**Table 4:** Ingredient formulation and chemical composition of TMR concentrate.

**Concentrate Ingredient Composition**	**% as Fed**
Maize	13.00
Beet pulp molassed	15.50
Soyabean meal 48% CP	30.00
Maize distillers	12.00
Acid buffer	0.70
Maize/Beet Min Balancer	2.50
Salt	0.50
Barley (rolled)	15.00
Rapeseed meal	7.50
Megalac	3.30
**Chemical Analysis**	**/kg as fed**
DM, g	874.90
UFL	1.02
UFV	0.99
Crude protein %	24.28
PDIN, g	169.26
PDIE, g	133.91
Starch %	18.10
Sugar %	6.92
Crude fibre %	6.10
Oil %	5.12
Ash %	7.99
Copper mg/kg DM	94.66
ME MJ/kg DM	11.16

Note: DM = dry matter, UFL = unité fourragère lait; UFV = unité fourragère viande, PDIE = sum of PDIA and the microbial true protein that is truly digested in the small intestine (PDIM) when energy is limiting; PDIN = sum of PDIA and PDIM when nitrogen is limiting.

**Table 5:** Average concentrations (Mean ± standard deviation) of raw milk metabolites (μM) measured during mid-lactation from cows fed diets consisting of total mixed ration (TMR), perennial ryegrass (GRS), or perennial ryegrass and white clover (CLV), as determined by ^1^H-NMR.

Metabolite (μM)	TMR	GRS	CLV	*p*-Value ^z^
3-Hydroxybutyric acid	34.06 (±10.63) ^b^	28.18 (±2.92) ^a^	24.63 (±4.75) ^a^	0.07 ^d^
Acetic acid	71.81 (±41.67)	41.47 (±10.17)	47.88 (±12.36)	0.12
Acetone	13.20 (±3.88) ^a^	11.66 (±1.22) ^a^	18.85 (±3.54) ^b^	<0.01
Alpha-Lactose	109,682.11 (±4835.98)	109,179.54 (±4189.15)	103,625.15 (±5267.57)	0.07
Aspartate	28.86 (±11.37) ^b^	28.63 (±13.11) ^b^	20.35 (±7.08) ^a^	0.33 ^d^
Betaine	90.15 (±25.33) ^a^	72.22 (±16.17) ^a,b^	64.41 (±31.44) ^b^	0.23 ^d^
Butyrate	29.22 (±5.72) ^a^	43.93 (±16.09) ^b^	35.91 (±16.47) ^b^	0.23 ^d^
Capric acid	20.66 (±5.75)	23.59 (±7.41)	19.89 (±5.74)	0.58
Caprylic acid	12.92 (±6.01) ^a^	23.01 (±8.03) ^b^	14.23 (±6.69) ^a^	0.04
Choline	214.22 (±58.37) ^a^	298.26 (±146.01) ^b^	310.54 (±77.69) ^b^	0.23 ^d^
*cis*-Aconitate	35.20 (±4.97)	35.13 (±6.74)	32.12 (±3.51)	0.55
Citric acid	4595.41 (±448.18)	4602.04 (±617.46)	4453.36 (±225.49)	0.77
Creatine	436.42 (±79.99)	432.95 (±84.67)	427.70 (±115.63)	0.98
Creatine Phosphate	60.35 (±28.96)	49.33 (±52.66)	38.24 (±39.15)	0.64
Creatinine	50.69 (±7.42) ^a,b^	53.41 (±9.12) ^b^	45.12 (±8.40) ^a^	0.23
d-Galactose	730.39 (±277.50)	901.88 (±624.82)	1023.29 (±936.59)	0.74
d-Glucose	331.24 (±84.17)	340.62 (±65.95)	387.43 (±91.21)	0.47
Dimethyl sulfone	11.67 (±1.59) ^a^	27.97 (±7.90) ^b^	46.41 (±12.72) ^c^	<0.01
Dimethylamine	14.60 (±2.77) ^a^	9.95 (±1.96) ^b^	9.40 (±2.30) ^b^	<0.01
Ethanolamine	84.09 (±27.64) ^a^	125.81 (±39.77) ^b^	116.79 (±23.05) ^b^	0.07 ^d^
Formate	117.40 (±1.66)	116.44 (±1.93)	115.20 (±2.75)	0.23
Fumaric acid	11.63 (±3.65)	11.20 (±4.36)	12.54 (±2.98)	0.77
Glucose-1-phosphate	52.60 (±27.92)	86.70 (±128.89)	28.31 (±27.81)	0.47
Glycerophosphocholine	617.18 (±83.87)	532.34 (±107.89)	531.67 (±240.19)	0.58
Hippuric acid	112.76 (±33.00) ^a^	227.93 (±38.90) ^c^	165.93 (±32.77) ^b^	<0.01
Isobutyric acid	15.87 (±20.47)	5.10 (±4.79)	14.45 (±15.29)	0.46
Isoleucine	5.15 (±1.64)	4.33 (±1.74)	4.20 (±1.53)	0.58
l-Acetylcarnitine	45.97 (±7.39)	51.71 (±12.86)	49.66 (±12.65)	0.64
l-Alanine	29.20 (±5.91) ^b^	27.45 (±5.67) ^b^	23.82 (±5.36) ^a^	0.27 ^d^
l-Carnitine	75.91 (±18.17)	81.36 (±20.84)	85.71 (±17.08)	0.64
l-Fucose	27.79 (±13.14) ^b^	21.03 (±14.33) ^a,b^	15.36 (±7.37) ^a^	0.23 ^d^
l-Glutamic acid	196.16 (±64.07)	188.62 (±77.08)	190.87 (±67.63)	0.98
l-Lactic acid	73.34 (±93.23)	47.31 (±26.19)	33.43 (±9.04)	0.49
l-Leucine	4.64 (±1.55)	3.80 (±1.31)	3.81 (±0.83)	0.47
l-proline	82.350 (±29.96) ^b^	55.55 (±12.68) ^a^	60.57 (±15.15) ^a^	0.07 ^d^
Malic acid	92.79 (±29.23)	83.66 (±28.83)	91.15 (±26.69)	0.78
Methanol	27.43 (±15.84)	23.64 (±7.16)	29.85 (±19.30)	0.74
Orotic acid	542.52 (±165.89)	581.43 (±134.08)	487.36 (±177.47)	0.59
Oxoglutarate	117.93 (±17.22)	116.48 (±18.59)	111.39 (±15.62)	0.75
*p*-Cresol	13.87 (±11.56)	13.39 (±10.02)	9.36 (±2.04)	0.62
Phosphorylcholine	72.12 (±59.74) ^b^	30.83 (±34.97) ^a^	44.14 (±32.01) ^a,b^	0.27 ^d^
Propionate	5.71 (±7.02)	7.58 (±6.92)	3.84 (±2.27)	0.58
Pyruvic acid	38.03 (±8.02)	36.52 (±14.70)	31.75 (±8.44)	0.58
Succinate	23.20 (±6.60)	19.73 (±3.18)	23.06 (±3.75)	0.41
Tyrosine	4.71 (±0.96) ^a^	8.11 (±1.97) ^b^	8.65 (±2.51) ^b^	<0.01
Urea	282.72 (±45.21) ^a^	221.68 (±102.60) ^a^	389.71 (±57.62) ^b^	<0.01
Uridine	16.07 (±4.91) ^b^	11.76 (±1.81) ^a^	11.78 (±2.37) ^a^	0.06 ^d^
Valerate	6.50 (±3.19)	6.47 (±2.84)	5.44 (±2.61)	0.74
Valine	11.27 (±3.98) ^b^	7.92 (±1.59) ^a^	8.55 (±2.14) ^a^	0.1 ^d^

^a,b,c^ indicates significant differences from Tukey Honestly Significant Difference pairwise test between treatments; ^z^ indicates *p*-value from ANOVA test; ^d^ Indicates attributes where significant differences were not found for both statistical analysis by ANOVA and Tukey Honestly Significant Difference pairwise comparison.

## Supplementary Materials

The following are available online at http://www.mdpi.com/2218-1989/8/2/27/s1, Figure S1: Partial least square discriminant analysis (PLS-DA) score plot of the rumen metabolome of lactating dairy cows fed diets consisting of total mixed ration (TMR), perennial ryegrass (GRS) or perennial ryegrass and white clover (CLV) as determined by ^1^H-NMR., Figure S2: Score plot of the partial least square discriminant analysis (PLS-DA) examining the effect of stage of lactation on the rumen metabolome of lactating dairy cows fed separate diets collected throughout each stage of lactation early, mid and late, as determined by ^1^H-NMR., Figure S3: Variable importance plot (VIP) which shows the compounds primarily responsible for separation of raw milk metabolomes from cows fed diets consisting of total mixed ration (TMR),perennial ryegrass (GRS) or perennial ryegrass and white clover (CLV) for the PLS-DA model., Table S1: Average concentrations of rumen metabolites (µM) measured in the rumen of lactating dairy cows fed diets consisting of total mixed ration (TMR), perennial ryegrass (GRS) or perennial ryegrass and white clover (CLV) throughout each stage of lactation early mid and late as determined by ^1^H-NMR.
