# Supplementary material for: Pasture Feeding Changes the Bovine Rumen and Milk Metabolome
Source: Metabolites. 2018 Apr 6;8(2):27. doi: 10.3390/metabo8020027 (PMC6027121; doi:10.3390/metabo8020027)
Supplement: Supplementary file 1 [file metabolites-08-00027-s001.pdf]

Supplementary Materials: Pasture Feeding Changes the Bovine Rumen and Milk Metabolome

Supplementary Table S1. Average concentrations of rumen metabolites (μM) measured in the rumen of lactating dairy cows fed diets consisting of total mixed ration (TMR), perennial ryegrass (GRS) or perennial ryegrass and white clover (CLV) throughout each stage of lactation early mid and late as determined by ^{1}H-NMR.

	**Early-Lactation**			**Mid-Lactation**			**Late-Lactation**			**SEM**
Metabolite (μM)	TMR	GRS	CLV	TMR	GRS	CLV	TMR	GRS	CLV	
2-Hydroxyisovalerate	4.57	8.48	8.53	10.71	5.74	5.50	5.89	4.29	8.44	0.74
3-Hydroxybutyric acid	6.80	7.83	9.22	7.55	10.85	12.63	23.98	7.35	16.05	1.56
3-Hydroxyphenylacetic acid	23.01	27.90	22.28	36.44	26.53	27.88	20.68	12.80	18.39	1.20
3-Phenylpropionate	779.59	717.96	554.52	761.80	704.73	759.75	696.33	476.13	588.32	19.66
4-Aminobutyrate	22.71	49.42	36.95	36.29	50.38	56.43	50.16	56.67	52.93	3.56
Acetic acid	56,450.48	55,371.36	57,153.50	59,611.64	55,080.78	59,988.47	49,825.50	54,056.92	57,825.81	834.44
Acetoin	20.89	20.64	21.65	25.69	22.45	25.61	29.64	21.00	28.60	1.10
Acetone	6.14	8.42	6.62	9.86	8.84	14.48	10.00	8.34	15.01	0.61
Adenine	29.17	31.67	42.77	29.28	35.09	37.18	20.86	11.59	11.68	2.06
Adenosine	3.27	6.70	3.14	6.29	6.40	8.43	4.09	7.24	3.67	0.60
Aspartate	115.08	96.53	122.66	144.89	158.90	217.53	199.76	92.07	103.86	8.45
Benzoic acid	25.42	30.23	28.39	25.50	27.81	26.28	20.71	20.91	27.73	0.82
Beta Alanine	7.38	11.08	7.21	14.03	27.64	29.24	33.88	16.00	15.88	2.00
Betaine	9.76	4.38	3.98	7.73	8.03	4.07	3.23	2.83	3.35	0.96
Butyrate	11,884.27	15,544.54	13,741.89	13,676.94	14,568.74	16,162.84	12,194.68	12,622.88	14,281.63	352.66
Cadaverine	53.46	128.58	109.88	82.03	93.26	107.44	111.32	67.68	103.96	7.00
Choline	20.93	11.82	13.28	20.48	16.65	15.76	36.26	15.57	8.73	1.62
cis-Aconitate	4.19	9.40	4.99	7.88	11.74	8.08	4.66	4.24	9.71	0.90
Citric acid	3.31	5.53	5.41	11.23	9.21	7.85	7.85	7.85	6.61	0.66
Creatine	7.71	6.76	7.08	9.90	8.13	6.92	8.46	5.90	6.79	0.66
D-Glucose	426.25	844.25	784.76	755.50	641.33	913.27	380.15	149.02	108.05	55.14
Dimethyl sulfone	6.04	19.23	37.43	3.03	14.60	36.46	2.43	16.59	26.23	2.00
Dimethylamine	1.68	3.14	2.08	8.06	1.82	9.18	3.02	1.97	3.13	0.96
Dimethylglycine	7.72	7.09	4.91	15.24	4.68	8.22	19.87	1.88	8.60	2.07
D-Maltose	124.21	58.00	43.83	31.74	29.01	39.19	57.68	16.20	17.03	6.30
Ethanol	12.53	112.58	27.92	21.71	37.59	42.91	42.65	27.70	24.28	8.88
Ethanolamine	28.92	32.07	34.83	37.34	37.35	43.58	21.87	15.35	10.03	2.07
Formate	115.52	114.01	116.01	121.81	113.23	119.66	118.16	117.49	118.62	0.60
Glycerol	209.23	220.06	219.03	286.20	270.45	294.14	269.32	236.48	233.55	6.28

Glycine	86.27	104.25	135.53	131.22	156.95	210.20	146.98	96.28	129.71	6.69
Hypoxanthine	161.40	169.98	213.58	190.70	203.38	213.12	161.39	104.28	111.55	7.38
Inosine	12.98	50.25	38.24	13.83	23.43	22.48	7.76	8.04	5.71	2.63
Isobutyric acid	808.14	745.82	775.18	818.37	859.50	1156.38	763.78	830.53	1061.15	21.81
Isoleucine	62.98	61.93	91.98	90.16	112.33	131.68	100.18	76.12	93.63	4.58
Isopropanol	16.71	23.59	26.03	19.85	26.48	63.61	17.92	16.67	36.89	2.57
Isovaleric acid	711.27	600.31	605.88	671.78	754.82	1187.49	667.28	748.80	1059.89	30.43
L-Glutamic acid	242.93	261.69	366.75	311.73	298.38	403.02	327.27	196.13	281.97	12.09
L-Alanine	139.00	189.28	211.52	206.31	231.38	306.74	195.89	148.08	198.38	8.72
L-Histidine	22.80	36.49	27.76	32.86	33.98	28.78	43.23	39.58	46.94	1.84
L-Lactic acid	14.46	20.63	18.28	26.22	18.61	36.30	51.30	48.99	27.90	4.02
L-Leucine	72.92	79.11	101.35	96.06	117.16	153.91	100.20	85.28	103.47	4.49
L-Lysine	143.95	170.81	176.06	202.61	240.94	323.59	102.05	109.71	118.18	12.56
L-Phenylalanine	42.16	43.90	50.09	58.58	68.03	78.82	53.72	40.00	44.38	2.53
L-Proline	77.17	67.54	95.11	86.45	107.08	161.58	89.36	66.88	68.31	6.43
L-Threonine	67.54	67.70	91.85	117.76	110.78	139.98	140.72	77.84	88.00	5.34
Methanol	10.76	9.68	11.35	10.78	10.37	13.90	19.68	9.01	9.08	1.17
Methionine	26.29	32.48	38.10	37.99	45.61	51.26	36.60	27.32	32.93	1.65
Methylamine	1.40	18.28	4.95	3.12	8.82	41.14	9.41	7.08	2.66	3.05
Nicotinate	24.98	35.88	42.51	31.36	30.60	37.12	20.97	19.21	28.18	1.33
O-Hydroxyphenylacetic acid	15.75	12.81	11.63	20.66	14.62	21.82	15.73	11.82	13.53	0.62
p-Cresol	55.29	43.48	37.74	57.63	75.38	110.54	62.23	78.99	107.27	4.00
Phenylacetate	202.23	159.84	157.23	194.39	318.03	536.45	202.11	308.47	449.38	20.18
p-Hydroxyphenylacetic acid	17.39	15.03	14.67	16.88	15.52	12.45	19.29	13.32	14.19	0.62
Propionate	17,764.88	20,803.31	18,280.66	19,344.51	17,120.38	17,982.38	14,248.39	16,330.55	18,159.29	463.30
Putrescine	64.55	89.79	59.37	65.59	53.28	48.96	46.63	26.36	28.37	4.77
Succinate	48.88	40.93	43.84	64.01	82.49	107.18	257.82	100.28	121.76	11.31
Trimethylamine	1.94	1.64	1.35	2.10	2.67	13.55	13.35	1.25	1.30	1.33
Tryptophan	6.21	7.33	8.28	7.97	9.41	10.69	7.34	5.42	5.59	0.37
Tyrosine	32.46	33.49	47.99	48.98	57.79	72.48	51.37	39.38	47.68	2.38
Uracil	225.92	287.28	350.22	285.07	364.46	373.89	90.48	140.81	168.73	14.57
Uridine	5.86	12.35	9.84	7.33	6.15	4.83	9.02	3.97	11.75	0.97
Valerate	1,020.82	1,715.66	1,141.45	1,201.26	1,212.65	1,611.75	869.19	842.36	1,231.22	62.85
Valine	71.25	71.60	92.41	106.05	118.54	191.78	103.48	81.25	102.18	6.42

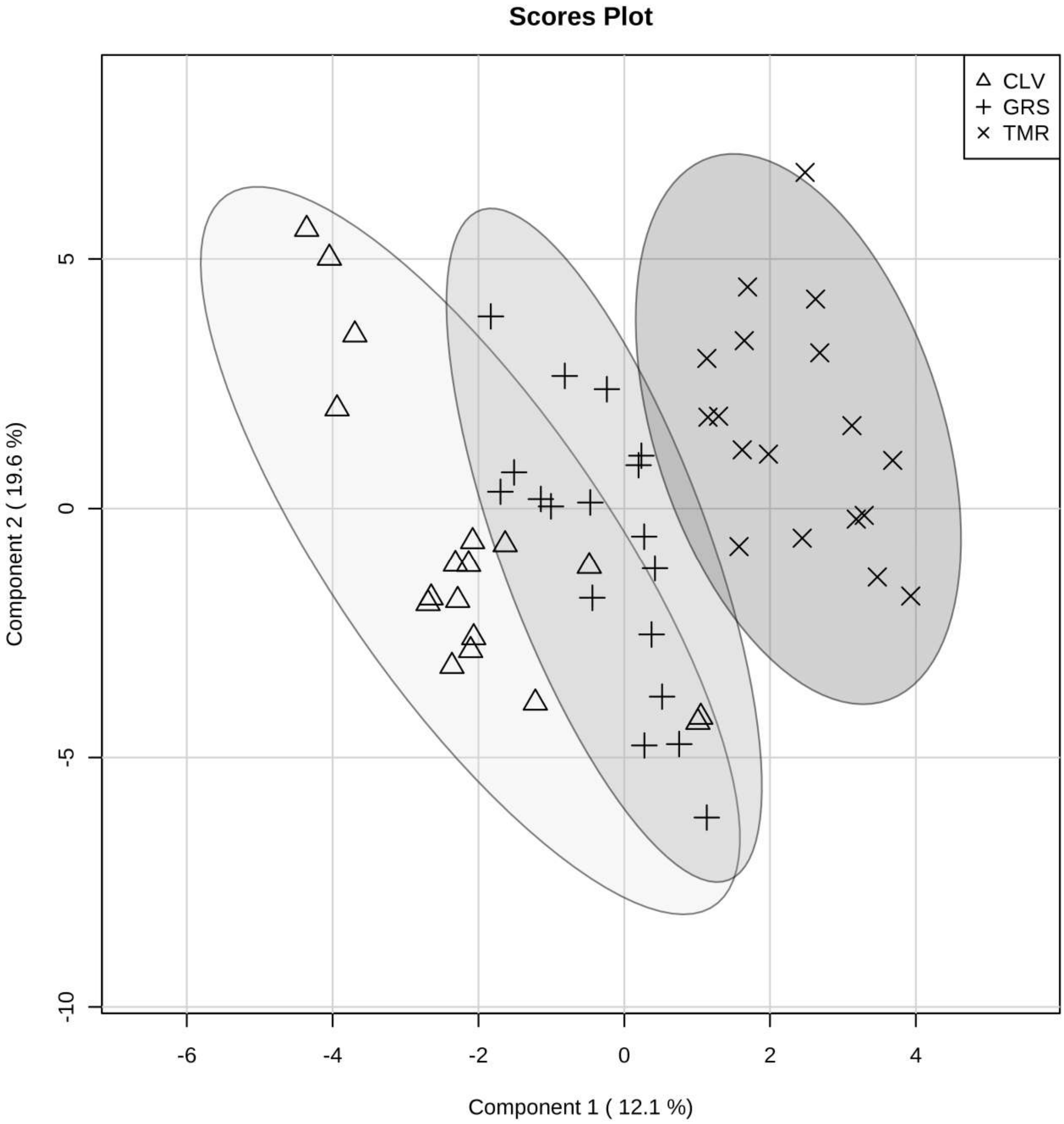

Supplementary Figure S1. Partial least square discriminant analysis (PLS-DA) score plot of the rumen metabolome of lactating dairy cows fed diets consisting of total mixed ration (TMR), perennial ryegrass (GRS) or perennial ryegrass and white clover (CLV) as determined by 1H-NMR. The shaded ellipses represent the 95% confidence interval estimated from the scores.

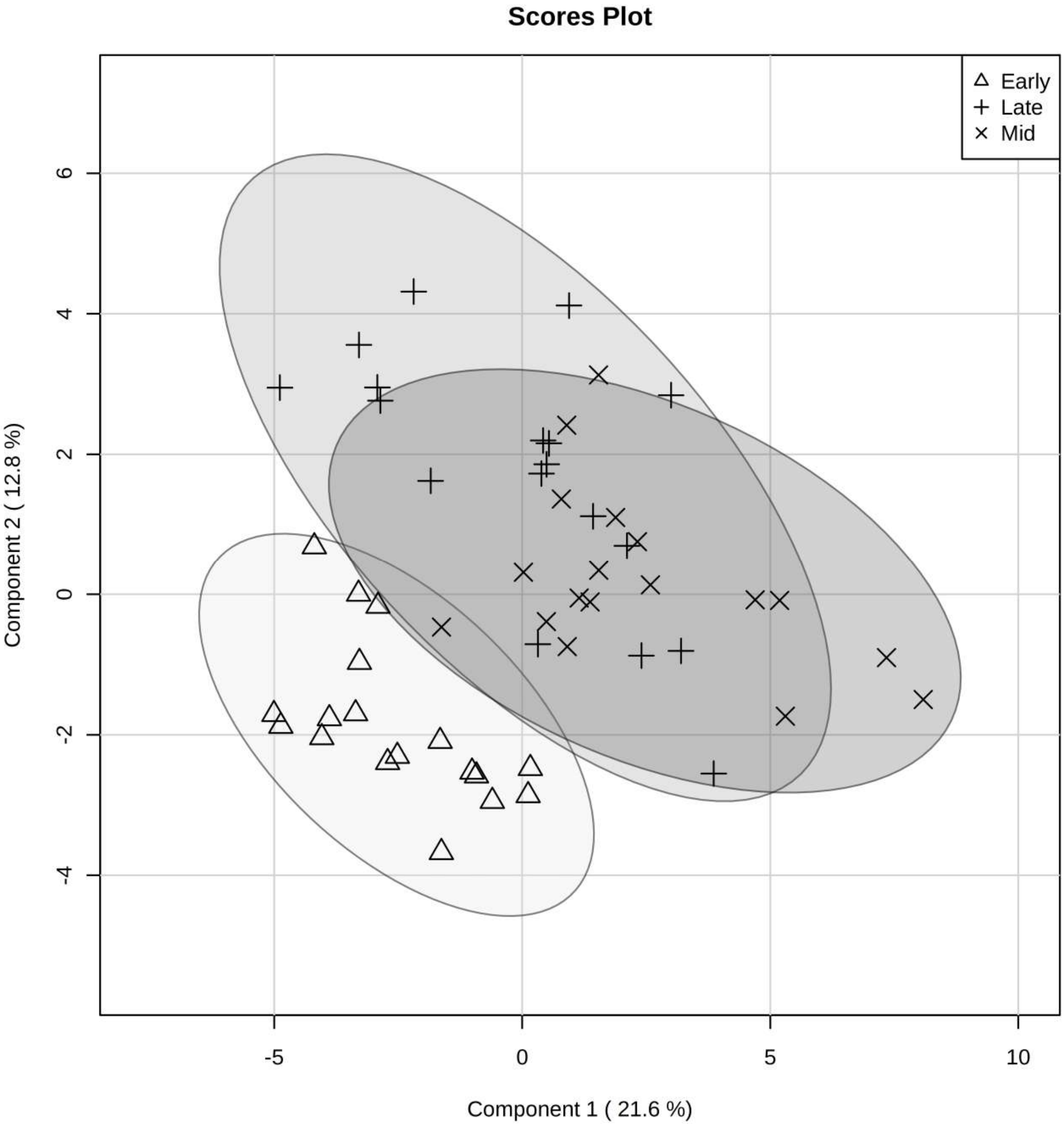

Supplementary Figure S2. Score plot of the partial least square discriminant analysis (PLS-DA) examining the effect of stage of lactation on the rumen metabolome of lactating dairy cows fed separate diets collected throughout each stage of lactation early, mid and late, as determined by ^{1}H-NMR. The shaded ellipses represent the 95% confidence interval estimated from the scores.

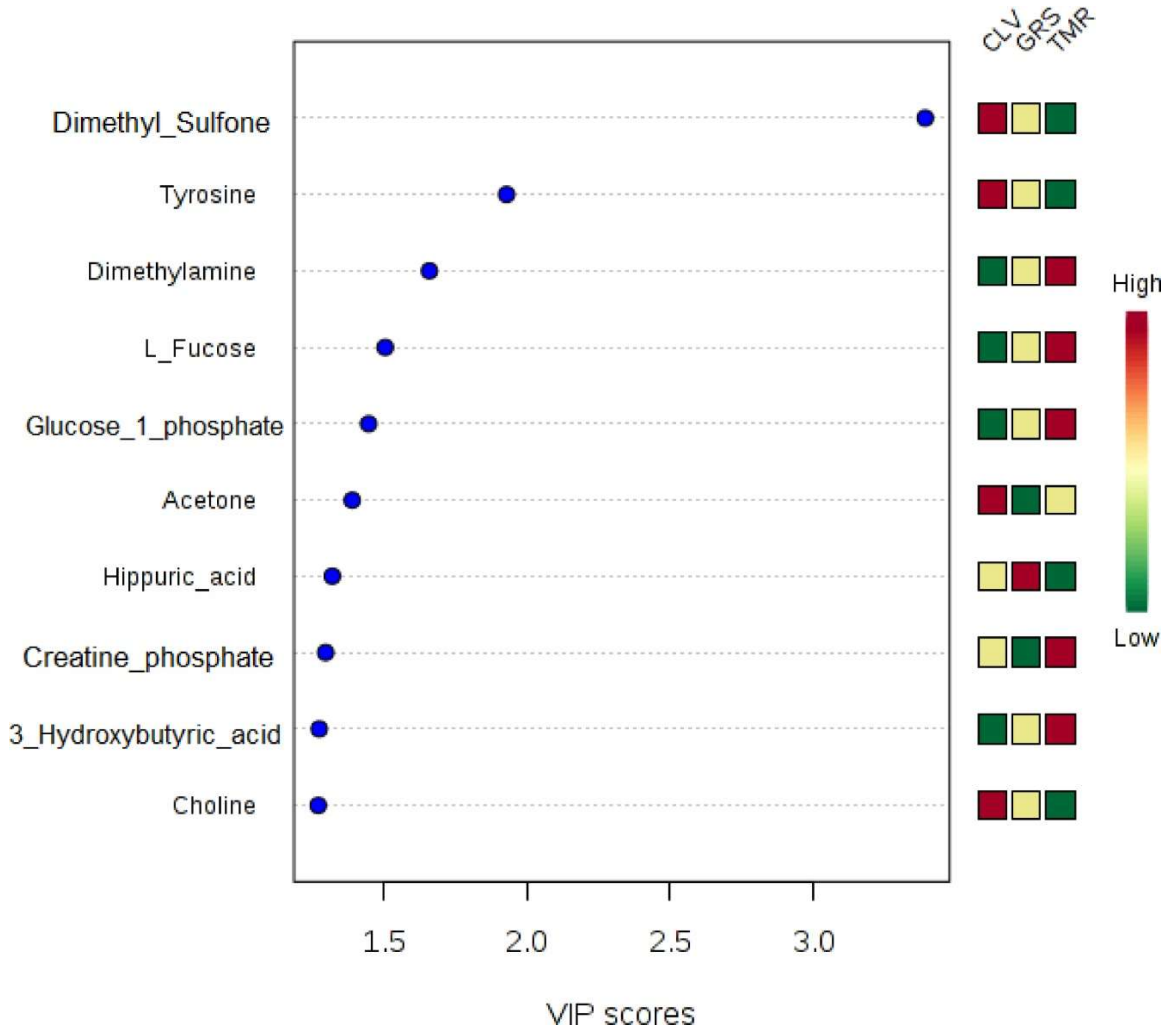

Supplementary Figure 3. Variable importance plot (VIP) which shows the compounds primarily responsible for separation of raw milk metabolomes from cows fed diets consisting of total mixed ration (TMR), perennial ryegrass (GRS) or perennial ryegrass and white clover (CLV) for the PLS-DA model.
